# Toward Prevention and Control of Type 2 Diabetes: Challenges at the U.S.-Mexico Border and Beyond^1^

**Published:** 2004-12-15

**Authors:** Barbara A Bowman, Frank Vinicor

**Affiliations:** National Center for Chronic Disease Prevention and Health Promotion, Centers for Disease Control and Prevention; National Center for Chronic Disease Prevention and Health Promotion, Centers for Disease Control and Prevention, Atlanta, Ga

Type 2 diabetes makes a compelling case study for public health action (1). The disease respects no boundaries. It is increasingly common — occurring in both developed and developing countries (2), in men and women, at earlier ages than in past decades, and in persons of every race and ethnic group, with a high prevalence in Hispanic/Latino Americans and in other minority groups, including non-Hispanic blacks, American Indians, Alaska Natives, Asian Americans, and Native Hawaiian and other Pacific Islanders (3). As noted by Martorell (4) and Saldaña(5), family history and genetic factors appear to further increase the risk for type 2 diabetes in Hispanic/Latino Americans. In the United States, the prevalence of diabetes was estimated to be 18.2 million people (6.3% of the population) in 2002 (3), with dramatic increases predicted in the future (6).

The determinants of type 2 diabetes are largely understood. Two of the most important risk factors, obesity and physical inactivity, are modifiable. The natural history involves progression from prediabetes, a condition in which blood glucose metabolism is abnormal (although not yet in the diabetes range), to the development of type 2 diabetes. The rate of progression from prediabetes to type 2 diabetes is between 3% and 10% per year (7). However, progression from prediabetes to diabetes can be prevented or delayed with sustained weight loss and increased physical activity (8,9). The magnitude of the change needed for primary prevention of type 2 diabetes is relatively modest: a 7% to 10% weight loss and sustained moderate physical activity, at least 30 minutes per day (10). Today, the number of adults with prediabetes in the United States is estimated to be at least 41 million (3).

Type 2 diabetes leads to devastating health and economic consequences for individuals, their families, and society. The most serious complications include blindness, kidney disease, lower-limb amputations, and acceleration of coronary heart disease and stroke (3). After type 2 diabetes is diagnosed, treatment requires an increasingly intensive and complex regimen to control glucose, blood pressure, and lipids, in addition to ongoing preventive care for the eyes, kidneys, and feet (11). Health care and complications attributed to diabetes are costly: in 2002, the total cost of diabetes was estimated to be $132 billion, $92 billion of which was spent on direct medical costs and $40 billion of which was spent on indirect costs, including disability, work loss, and premature mortality (12). Clearly, ongoing access to high-quality health care is a paramount concern for preventing complications and death from diabetes. Such care is expensive, and much of the cost of drugs and supplies is not reimbursed, even for those with insurance coverage (13). While it is improving, the quality of clinical care for people with diabetes still falls short of established guidelines (14). Because of continued increases in the prevalence of obesity, the outlook for the future is ominous — the health system will likely be overwhelmed by type 2 diabetes (15).

The population groups at increased risk for diabetes, including Hispanic/Latino Americans, suffer a disproportionate burden of disease, further exacerbated by poverty and lack of access to health care (3,16). What public health responses are likely to be effective in reducing the present and future consequences of type 2 diabetes in population groups, such as people living along the U.S.-Mexico border? And, how long will it take to begin to turn the tide?

As detailed by Cohen et al in the series of articles from the Border Health Strategic Initiative, the solution to type 2 diabetes control must begin in the community (17). Extensive dialogue is a first step in engaging communities and identifying the priorities for community action. The papers by Cohen and associates demonstrate how communities and researchers can — and must — collaborate to assess targets for intervention and develop sustainable solutions to control type 2 diabetes. Insights gained from these interventions also can guide the development of effective community-based approaches for primary prevention of type 2 diabetes. Community-based participatory research and mobilization are critical to create the evidence base for elimination of health disparities, as shown in a recent compendium of papers describing the experience of Racial and Ethnic Approaches to Community Health (REACH) 2010 communities (18).

But having evidence is not enough. Improving the public's health will require rapid translation and dissemination of effective, community-based strategies for diabetes prevention and control and the commitment to sustain and reinforce these interventions (19). As shown by this promising initiative (17), collaboration across and within national and state borders and communities will be essential and must involve the entire community: where people live, work, play, and go to school. Improved clinical care alone will not be sufficient. One strategy now being implemented uses the essential public health services as strategic levers to strengthen the public health response to diabetes (20). Development, implementation, and evaluation of such strategies are needed urgently. We anticipate that publication of the papers by Cohen et al, which describe many challenges and some successes, will inspire readers of *Preventing Chronic Disease* to share their own lessons learned and promising approaches for public health action to prevent and control type 2 diabetes.
